# Women’s experiences with traditional medicine for the treatment of cervical cancer: a qualitative study

**DOI:** 10.1186/s12906-026-05344-z

**Published:** 2026-03-13

**Authors:** David Ayangba Asakitogum, Lillian Akorfa Ohene, Lydia Aziato

**Affiliations:** 1 Department of Adult Health, School of Nursing and Midwifery, University of Ghana, Legon, Ghana; 2 Department of Public Health Nursing, School of Nursing and Midwifery, University of Ghana, Legon Accra, Ghana; 3 Vice Chancellor, University of Health and Allied Sciences, Ho, Ghana; 4 Ataribuno Cervix Foundation, Bolgatanga, Ghana

**Keywords:** Cervical cancer, Complementary and alternative medicine, Experiences, Herbal medicine, Patients, Qualitative research

## Abstract

**Background:**

Over 60% of patients with cancer in sub-Saharan Africa used traditional medicine for cancer treatment. The common predictors of traditional medicine use include being a female. However, there is limited empirical information about the experiences of women with traditional medicine usage for the treatment of cervical cancer. The current study explored the experiences of women who used traditional medicine for the treatment of cervical cancer in Accra, Ghana.

**Methods:**

An exploratory qualitative research design was used in this study. A semi-structured interview guide was used to interview 12 women to gain insight into their experiences with traditional medicine for cervical cancer treatment. The data collected were analyzed using content analysis in which major themes and subthemes were generated. These findings were supported with verbatim quotes from the participants.

**Results:**

One overarching theme emerged with six (6) corresponding sub-themes. 1. Traditional treatment for cervical cancer (subthemes: treatment without a diagnosis, herbal medicine for cervical cancer, prayer for healing cervical cancer, insertion of substances into the vagina as treatment for cervical cancer, cost of traditional treatment, and traditional treatment outcomes).

**Conclusion:**

Women used herbal preparations, prayers, and insertion of substances into the vagina for the treatment of signs and symptoms of cervical cancer. The women received herbal prescriptions from herbalists or traditional medicine practitioners without a definite diagnosis and consulted pastors in prayer camps after their cervical cancer diagnosis for healing prayers. These findings have implications for cervical cancer treatment and patient safety in Ghana.

**Supplementary Information:**

The online version contains supplementary material available at 10.1186/s12906-026-05344-z.

## Introduction

Cervical cancer is a public health issue affecting women in Ghana. It is the second cause of cancer-related deaths among women in Ghana [1]. In 2023, the crude incidence rate of cervical cancer was 18.3 with about 1699 deaths of the women in Ghana [2]. Cancer patients in low-income countries such as Ghana use complementary and alternative medicine (CAM) [3]. Herbal medicine (HM) is synonymous with these complementary medications in Ghana [3, 4]. Traditional medicine (TM) combines HM and CAM and refers to the use of healthcare practices or products that do not fall under the purview of mainstream medicine (biomedicine) and which have not been fully incorporated into the country’s dominant health system [5].

The use of TM varies across the globe. In a systematic review and meta-analysis of 152 studies with over 65 000 patients with cancer from 18 countries in Europe, the United States of America (USA), Canada, and Australia, the mean prevalence of CAM use was 40% - the highest from the USA (50%) and the lowest in Italy and the Netherlands (22%) [6]. Recent quantitative studies reported 43% (*n* = 706) of CAM use in Norway [7], 36.6% (*n* = 530) of HM use in Morocco [8], and 39.1% (*n* = 350) of CAM use in Trinidad and Tobago [9] among cancer patients. Common types of CAM used in cancer patients were herbs [7, 9], self-practices (i.e., relaxation, yoga, and meditation) [7], and spiritual therapy (e.g., prayer) [9, 10]. Some common predictors of HM and CAM use included being female [7–9], social norms of relatives [8, 9, 11], and perceived scientific and socioeconomic barriers [8]. This evidence shows that women are more associated with HM and CAM use. However, there is limited empirical information about the experiences of HM and CAM use among patients with cervical cancer.

Between 70% and 90% of HM and CAM users self-perceived improvement in their cancer symptoms [7, 9, 12]. In a review in China [13], Chinese traditional medicine (CTM) was reported to enhance the efficacy of chemotherapy and radiotherapy and diminish the side effects and complications of these therapies in cancer patients. The review provided evidence that CTM (e.g., TJ-41, Liu-jun-zi-tang, Ginseng, Huang-Qi, Huachansu injection, etc.) suppressed tumor progression, relieved surgery complications, increased sensitivity of chemotherapy and radiotherapy, and improved cancer patients’ immune system [13].

In sub-Saharan Africa, in two scoping reviews [14, 15], the median prevalence of HM and CAM use among cancer patients was 60% (range: 13%-80%). In these reviews, the use of herbs, healing prayers, and body massage were identified as CAM practices. The main reasons reported for the use of HM and CAM include the desire to get rid of cancer symptoms (e.g., pain), treat side effects of chemotherapy, improve immunity, and the need to improve physical and psychological well-being [14–16]. However, many patients with cancer in sub-Saharan Africa do not disclose HM and CAM use to their doctors in the hospital [15]. The non-disclosure of HM and CAM use among patients with cancer may impede the efforts made in cancer education, particularly health education that targets the prevention and control of cervical cancer.

In Ghana, two qualitative studies [17, 18] assessed patients on HM use and/ or spirituality, and another two studies [3, 4] explored traditional herbalists and cancer management practices. The traditional herbalists in Ghana believed that the use of spiritual therapies (i.e., prayers or divination) and herbal remedies are effective for cancer cure [3, 4]. Similarly, the facilitators of herbal use among adult patients in Ghana were identified as perceived effectiveness of and personal preference for HM while the barriers against HM use were negative perception about HM, poor vending environment, and poor knowledge of vendors [17]. Patients with cervical cancer in Ghana used spirituality as a coping strategy, a sense of hope, and the will to live with the cancer [18]. They believed in and trusted herbalists and would consult herbalists for healing [19]. However, no study was found that explored patients with cervical cancer experiences with TM use. Understanding how these patients use TM (e.g., types, forms, routes, etc.) to treat cervical cancer is imperative for the development of cervical cancer prevention and control strategies. Therefore, the objective of this study was to explore women’s experiences with TM use for cervical cancer treatment in the Accra metropolis, Ghana.

## Methods

### Research design

An exploratory-descriptive qualitative design was adopted in this study to explore the subjective experiences of women with TM use for cervical cancer treatment. This research design ensured that the researcher probed the women’s experience of the phenomenon and described in detail the findings [20]. The focus of the design was to gain insight into the women’s experiences during the use of TM to treat cervical cancer.

### Study setting

The study was conducted in the Accra Metropolis. Accra Metropolis is the seat of the Government of Ghana and the national business City. Because of the cosmopolitan and business nature of Accra, different ethnic groups of people reside in the metropolis. The most popular ethnic groups living in the Accra metropolis include Akan, Ewe, Fante, and Hausas, as well as the indigenes (i.e., Ga and Ga-Dangme). Many of these indigenous groups of people believe in and practice various forms of traditional medicines complementary to hospital system care. The cervical cancer screening center in the Greater Accra Regional Hospital (GARH) was used as an outlet for the recruitment of the participants for the study. The GARH is the most popular cervical cancer screening and diagnosis referral center in Ghana. The center receives patients from all parts of Ghana for cervical cancer diagnosis. The Accra Metropolis was used as the setting because patients who received cervical cancer diagnosis and had used TM to treat the cancer could easily be traced and contacted.

### Target population and sampling

The target population for this study was all women who were referred to the cervical cancer screening center at the GARH for cervical cancer diagnosis. Women who received a cervical cancer diagnosis at the center, had used TM to treat the cancer, aged 18 years or older, and reported from the Accra Metropolis were included in the study. Women who reported from other parts of Ghana were excluded because their locations could not easily be traced. Those who were too ill (defined as being on hospitalization) were excluded. Women who met the inclusion criteria willingly accepted to be part of the study and consented were recruited as participants through purposive sampling. Twelve (12) out of 140 patients who met the above inclusion criteria were interviewed, and data reached saturation. Data saturation is when no new information is coming from the interviews with participants in a study, which ensures that adequate and quality data are collected [21]. On the tenth interview, no new information was realized, and two more interviews were conducted, and data reached saturation.

### Data collection procedures

The first author collected data for this study. Patients diagnosed with cervical cancer were identified through the institutional register by a staff of the cervical cancer screening center. The staff at the center contacted the potential participants on the phone and explained the purpose of the study to them and referred those who wanted to learn more about the study to the researcher or their contact be giving to the researcher to contact them. Interested participants contacted the researcher and shared their contact numbers and/ or home addresses with the researcher to contact and schedule interview dates, time, and place of interview. The purpose of the study was explained to the participants who agreed and met the inclusion criteria. Face-to-face interviews in English and Ashanti Twi were scheduled and conducted from November 2017 to May 2018. Only two participants were interviewed in Ashanti Twi, seven were interviewed at GARH, and five were interviewed at their workplaces in an office. Four participants initially signed up but later refused the interview because their husbands did not consent. Each interview session lasted between 45 and 60 min. The interviews were based on open-ended questions about the use of TM to treat cervical cancer. The questions were developed in the form of a semi-structured interview guide by the authors according to the objectives of the study (Supplementary Table_1). The questions were pretested using two clients at the GARH but were not included in the analysis. Participants’ responses were probed appropriately, and the interviews were recorded with the participants’ permission. Field notes on participants’ gestures and researcher reflections were taken alongside the interviews to consolidate responses and feelings respectively. No compensation was paid to the participants except for those who came to GARH purposively for the interview received reimbursement of lorry fare (Ghc15 ($3) maximum).

### Data management and analysis

Data collection and analysis were carried out concurrently. Content analysis principles and procedures were used for the data analysis. Two of the interviews were first translated from Ashanti Twi into English and transcribed by the second author, who understands and writes the Twi language. All the interviews were transcribed verbatim. All authors read through the transcripts repeatedly to gain the meaning of the responses. The ideas were first identified and coded. Similar codes were re-grouped to form subthemes. The subthemes were then used to generate major themes. The resulting thematic structure served as the organizing framework for the results of the study [22]. The data was managed by manually sorting the codes and quotes under respective themes and subthemes. These were reviewed by all the authors and a consensus was reached after the reviews. The transcripts and audio recordings were saved on the researcher’s personal computer in identifiable folders with security code/password to make them inaccessible to any other person, according to the Data Protection Act.

### Rigor

The study included participants who were diagnosed with cervical cancer and had used TM to treat cervical cancer in the Accra Metropolis. All the participants had used one type or form of TM. This ensured data homogeneity. The data collection and analysis were done concurrently which ensured member checks and follow-up on emerging themes. Field notes and reflective journals were also kept for audit purposes. The use of the same interview guide by the same interviewer also ensured the rigor of the study.

### Ethical considerations

The study received ethical clearance from the Ghana Health Services Ethics Review Committee–GHS-ERC 011/10/17 and Noguchi Memorial Institute for Medical Research Institutional Review Board – NMIMR-IRB-022/17–18 of the University of Ghana. Institutional approval was granted by the GARH to use the cervical cancer screening center register to identify participants for the study. Participants were told that participating in the study was voluntary and that refusal to participate would not in any way affect their healthcare at the facility. Individual informed consent forms were obtained from all the participants who willingly agreed to participate.

## Results

### Demographic characteristics of participants

Twelve (12) participants were recruited into this study. The mean age of participants was 53.7 (range: 35–68). Most of the participants attained a basic level of education. All the participants were once married (see Table 1 below for details). To ensure confidentiality, the participants were represented by codes (e.g., C1, C2, etc.)

**Table 1:** Demographic characteristics of participants

Code	Marital Status	# sexual partners	# children	# clinic/ hospital visited	Level of Education	Length of cancer diagnosis	Family planning	Traditional Treatment
C1	Married	5	2	6	Tertiary	2 years	Yes	Herbs
C2	Divorce	5	2	5	Tertiary	5 years	Yes	Herbs
C3	Divorce	3	4	8	Tertiary	4 years	Yes	Dietary
C4	Widow	3	4	7	Secondary	18 months	No	Herbs
C5	Divorce	10	4	10	Secondary	16 months	No	Herbs Prayer Insertion
C6	Divorce	1	3	9	Basic level	6 years	Yes	Prayer
C7	Married	3	8	8	Basic level	2 years	Yes	Prayer Herbs
C8	Married	1	3	6	Basic level	3 years	No	Prayer
C9	Married	1	3	8	Secondary	4 years	Yes	Prayer
C10	Married	2	3	8	Basic level	5 years	Yes	Prayer
C11	Married	4	3	6	Tertiary	3 years	Yes	Prayer Herbs Insertion
C12	Widow	5	5	10	Basic level	4 years	Yes	Prayer Herbs Insertion

### Thematic findings

One overarching theme emerged from our analysis of the experiences of women with TM for cervical cancer treatment. 1. Traditional Treatment for Cervical Cancer with 6 subthemes (subthemes: treatment without a diagnosis, herbal medicine for cervical cancer, prayer for healing cervical cancer, insertion of substances into the vagina as treatment for cervical cancer, cost of traditional treatment for cervical cancer, and traditional treatment outcomes for cervical cancer). The sections below elaborate on these subthemes in detail.

- 1. Traditional Treatment for Cervical Cancer

The women in this study indicated that they have used herbal medicine, prayers, and the insertion of substances into the vagina for the treatment of cervical cancer. The women further expressed that the treatment was readily available and that there was no specific diagnosis made before the commencement of treatment.

- 1.1 Treatment without Diagnosis

The study showed that when herbalists and/or friends were confronted with cervical cancer complaints, the signs and symptoms presented became the basis for the prescription of treatment.

*“…I discussed with them* (friends) *what I was going through like the discharges. They didn’t know it was cervical cancer. They knew it was itching*,* discharges*,* and abnormal bleeding from my private part. And they said when people have those* things (signs and symptoms) *that are the treatment for it…so I went to her* (herbalist) *for it” (C1).*

*“When I told my friends about how I was feeling*,* they advised that it was just a sore if I take that medicine* (herbal) *it will go. So*,* I started all that for one year and as I was taking it I saw that it is rather becoming severe” (C5)*.

The data gathered also revealed that close friends and herbalists used previous experience to prescribe treatment for cervical cancer.

*“Some of them* (friends*) said they knew of ladies who have similar problems and have used them* (herbs*) and they* (ladies who used herbs for similar problems*) are fine*,* so I used it too…” (C1)*.

*“You know*,* she* (herbalist) *has also gone through that herself*,* she had that pain when she was in school and was flown to Liberia and there it didn’t work. So*,* she came back to Ghana*,* and she got that herb. And since that time*,* she has been treating all these diseases. She has more experience in handling women’s problems” (C11)*.

- 1.2 Herbal Medicines for Cervical Cancer

Nine (9) out of twelve (12) participants used herbal preparations in the form of a fluid (sap) to treat their cervical cancer. Drinking, bathing, and douching were how the sap was delivered into the body for healing.

*“.Tree leaves*,* branches*,* different (3x) flowers that they brought. I boiled them before I use. I used some to bath*,* some to wash my private part*,* and I drink too” (C1).*

*“.He* (herbalist*) gave those things* (herbs) *to me and instructed me to boil them for 3–5 minutes*,* sieve them*,* and drink them like water. So*,* I did it and drank twice a day*,* morning*,* and evening*,* that is all” (C4).*

The women stated that they consulted close friends and herbalists who then prescribed herbs and other substances to be used as a treatment for cervical cancer.

*“One of my friends took me to a man who cooks this herbal medicine. He gave me the roots*,* not the leaves to come home and cook it and use” (C5).*

*“It was my sister’s friend who brought the herbs from a certain herbalist. We were convinced that it would work since it has worked for others. I never knew my situation was different” (C12).*

The women identified the herbal preparation processes as, boiling the herbs and sieving the sap before use.

*“…I was drinking leaf tea and some other leaves…I boiled it and sieve the water into a water container and drink it” (C2).*

*“No*,* it was not only the balm that I used. She* (herbalist) *also showed me some roots to buy and boil it*,* sieve it and drink as well. She* (herbalist) *said combining these will help to kill the disease fast” (C11).*

- 1.3 Prayer for Healing Cervical Cancer

All the participants believed that God had healed them through their prayers. They stated that the prayers were said purposely for healing cancer.

Some of the participants believed that they were living by the grace of God and its only God who had healing power.

*“…I came back home for two months after the diagnosis. I did not go because I was praying… I have been living by God’s grace*,* I pray a lot I know God is my healer*,* so I pray a lot…” (C5).*

Some of the participants consulted Pastors at prayer camps for prayers and healing services for their cervical cancer.

*“The first time they told me that I have the disease; I didn’t have money to do the treatment. So*,* I went to a prayer camp trusting that with the prayers the cancer would go. The pastor prayed for me several times for a year*,* but the cancer was rather increasing” (C12).*

*“I went to my pastor for prayers… it is just like my normal prayers*,* but it’s said with the purpose of healing my cancer. I told my pastor that pastor I get cancer pray for me. So*,* it is my pastor who brought a professor at some time*,* and they sent me to the first doctor” (C6)*.

Many of the participants stated that they fasted and prayed for the healing of their cervical cancer.

*“I have not sent it to church*,* but I always pray when I’m in the house. Sometimes I also fast and pray. I prayed that God would not let them operate on me. He* (God*) should operate on me in the spirit and heal me so that I will give Him the thanks” (C7).*

*“Hmm*,* I became tired of fasting and prayer for this thing* (cervical cancer*) to go away. But now I’m grateful to God that He has healed me through the hands of the doctors” (C11).*

However, one participant prayed to God to avert surgical and treatment mistakes likely to arise from the cancer treatment.

*“We are human beings and mistakes can happen*,* so I asked God to help the doctors to remember everything they learned about the treatment of this disease and treat me without mistakes. I was having hope*,* but I pray to God that nothing bad should happen during the operation” (C8).*

- 1.4 Insertion of Substances into the Vagina as Treatment for Cervical Cancer

The study revealed that some of the women had inserted ‘balm’, penicillin ointment, and other substances into the vagina to treat cervical cancer.

*“So*,* I will sit in hot water and also insert something like penicillin ointment to cure it” (C5).*

*“I met this woman who was selling medicinal oil something like a balm. She told me it’s good for treating vagina infections and sores. I bought them and usually*,* after a bath*,* I will insert it into the vagina” (C11).*

*“I consulted an herbalist in Nima who gave me some things to always insert into my private part in the evenings” (C12).*

However, one participant mentioned that the insertion of penicillin ointment was meant to treat the sores on the cervix.

*“…I inserted something like penicillin ointment to cure it. I was thinking like it is sore if I insert the ointment*,* the sore will go” (C5).*

Apart from the use of herbs, prayers, and substance insertions, one participant indicated that she used fruits, vegetables, and some iron supplement drinks to treat the cancer.

*“…What you must stop eating and what you must be eating. I must eat green vegetables*,* nuts*,* coconuts*,* groundnuts*,* and pomegranates*,* I tried to drink coconut water 2x a day*,* ‘atta twe’* (tiger nuts*) I chew it even when I’m in a car…A certain fruit in Akan called ‘aluguitugui’ (local apple) I eat it and sometimes blend it and drink it… I squeeze lemon in warm water and drink it every day…I believed that these things helped me” (C3)*.

- 1.5 Cost of Traditional Treatment for Cervical Cancer

Most of the participants considered the cost of herbs, prayer, and ‘balms’ to be inexpensive.

*“They showed me some different (2x) tree leaves to cut and boil. It was at no cost to me” (C1)*.

*“I have to go to the hospital and pay some huge amounts*,* but the herbal medicine is not like that” (C5).*

*“Ooh no*,* the pastor didn’t charge me for anything. But I do my normal church offerings” (C6).*

One participant who used food and dietary products considered the cost of traditional treatment to be expensive.

*“It was in a big bottle and that day it cost me about Ghc700. In all*,* I spent about GHc1500 on those supplements and the drinks. This excludes vegetables and fruits which have now become my diet and the diet for the family… it’s a bit expensive” (C3)*.

- 1.6 Traditional Treatment Outcomes for Cervical Cancer

In this study, four (4) of the women expressed a better health status for TM usage.

*“…. So*,* I think changing my eating lifestyle had helped me to be cured” (C3).*

*“Ooh*,* I am ok ‘appears excited’. Yes*,* since that time till now*,* I am ok. So*,* I can eat properly now*,* first I couldn’t even take water*,* which was a problem*,* but going through that (herbal treatment) I’m ok. I don’t feel any pain. At first aaii*,* I can’t even sit up and talk to you. The pain is so severe and vomiting but now is gone” (C4)* .

“*The herbs medicine has helped me.because you know for therapy* (radiotherapy), *I’m not drinking it*,* I’m sure that drinking the herbs*,* it kills the internal” (C5).*

Two (2) of the women reported that their health status worsened because of the use of traditional treatment for the cancer.

*“It is because the herbs did not help me. It was becoming worse. I said to myself that no*,* I have used this herb for some months now and there are no changes; I must go to the hospital” (C1).*

*“To me*,* the herbs and the prayers did not help me. I was becoming weaker and weaker. I only thank God that the doctors at GARH were able to know that it was cancer” (C11).*

## Discussion

This is the first study to explore women’s experiences with TM usage for the treatment of cervical cancer from the patient’s perspective. The findings indicate that the women received traditional treatment without a definite diagnosis of cervical cancer from the herbalists. The main form of TM the women used for the treatment of cervical cancer was HM. The study also found that the women consulted pastors and used prayers for the healing of cervical cancer. Some of the women also inserted substances into the vagina as a treatment for cervical cancer. These findings highlight the role of herbalists and pastors and the importance of HM and prayer in the healthcare system in Ghana and beyond, particularly in the care and prevention of cervical cancer.

As the first study to explore women’s experience with TM usage for the treatment of cervical cancer, a direct comparison of the study findings to extant literature is not possible. However, the discussion situates the findings in the context of CAM for heterogeneous types of cancer care and management and/ or in a broader perspective of TM. All the women consulted herbalists or traditional medicine practitioners (TMPs) first before their cancer diagnosis. Herbalists/TMPs are believed to perform a variety of roles, including the ability to address the supernatural dimensions of the cause of diseases and provide spiritual and social support that promotes the total healing and well-being of patients [23, 24]. There is also perceived greater privacy and absolute trust for herbalists/TMPs and/ or in the access to TM for cervical cancer as against mistrust for healthcare providers [19, 23]. Evidence also suggests that CTM is effective for the cure of cancer and possesses the ability to decrease the side effects of chemotherapy and radiotherapy in patients with cancer [13] as well as in cervical cancer [25]. TM is also readily available [23, 26]. Inadequate availability of cancer treatment health facilities, high cost of cancer treatment, and longer patient travel distances are barriers against access to conventional cancer treatment in sub-Saharan Africa [8, 23]. These are the compelling reasons that continue to attract patients with cancer to seek TM first, as confirmed in this study.

The women expressed that they were not given a definitive diagnosis of cervical cancer in their consultation with the herbalists. Rather, the treatment the women received was based on their presenting symptoms. It was also clear from the data that the herbalists drew on previous experiences and success stories for the prescription of treatment for new clients. While further studies are recommended to confirm the inability of herbalists to give a definitive diagnosis of cervical cancer, it is acknowledged that herbalists were not interviewed in this study, and therefore the women might not be privy to herbalists’ diagnostic system. However, herbalists or TMPs in Ghana relied on patients’ presenting symptoms (i.e., visible masses, fungating lesions, ulceration, and bleeding) and physical examinations, as well as divination to be able to prescribe treatment for patients with cancer [3]. Since giving diagnostic information to patients is critical in the healthcare industry [27], the study recommends herbalists give concrete diagnoses and inform patients with cervical cancer of the diagnosis.

Consistent with previous quantitative studies in heterogeneous types of cancer [7, 9], in this study, the women used herbal preparations in the form of fluid (sap) for the treatment of cervical cancer. While no study attempted to describe how herbs are used in the treatment of cervical cancer, in this study, the medicinal substances for the herbal preparations were prescribed by herbalists or TMPs in the form of herbs, roots, branches, leaves, and flowers. These medicinal substances were either supplied to the women or the women were asked to buy them in the market. Based on the guidelines for preparation and the prescription for use, most of the women prepared the medicinal sap themselves. Some of the women described the herbal preparation for the treatment of cervical cancer as cooking or boiling the prescribed medicinal substances for 3–5 min, sieving and cooling the sap, storing the sap in a clean container, and drinking them as water or tea for up to 24 h. For others, after sieving and cooling the boiled medicinal substances, the sap was used to either bathe or wash the vagina (douching). The study identified drinking (oral), bathing (skin), and douching (vagina orifice) as the routes for administration of TM for the treatment of cervical cancer. The active involvement of the women in the herbal preparation for the treatment of cervical cancer in this study may be an incentive for self-trusting and empowerment for the women. In two qualitative studies on traditional herbalists and cancer management practices in Ghana [3, 4], the herbalists reported using herbs, roots, shrubs, tree bark, leaves, flowers, parts, and fruits to treat various types of cancers. The herbalists also reported consumption (drink) and topical application as methods of delivery of herbal products in the treatment of cancer [4]. What remains unclear are the dosages of the herbal products and their safety for use either alone or in combination with conventional cancer treatment for cervical cancer. The study recommends further investigations on the identification of the prescribed medicinal plants and analysis of their anti-cancer properties for the treatment of cervical cancer.

Consistent with previous studies in heterogeneous types of cancer [9, 10, 28, 29], the women in this study used spiritual therapy for the treatment of cervical cancer. The reasons for engaging in spiritual therapy vary among patients with cancers. For instance, in the study of American Indian women cancer survivors [10], prayer and spirituality were reported to have connected the women to their families and faith communities. In the studies among women with cervical cancer [18, 29], spirituality was used as a coping strategy which helped patients accept and live with the cancer. In this study, the women used and perceived prayer as a treatment for cervical cancer. Some of the women purposely contacted pastors in prayer camps to pray and invoke healing for the cancer. Other women reported fasting and praying continuously in their rooms while waiting for God to operate on them and remove the cancer. While it is acknowledged that prayer and spirituality bring psycho-healing to cancer patients [10, 18, 29], these practices should not cause delay or interferes with medical therapies as was pointed out in our previous study [27]. These findings demonstrate the inseparable nature of God in the lives of these women in times of difficulty in seeking comfort and understanding through faith. The findings further identified pastors as stakeholders in the development of strategies for the prevention of cervical cancer.

In addition, one unique finding was that the women inserted substances (e.g., “balm” (burn crushed herbs and roots and mix into Vaseline), penicillin ointment, etc.) into the vagina as a treatment for cervical cancer. These insertable products were recommended to the women by the herbalists and the women’s personal friends who had used these insertable products to treat vagina infections. The purpose of the insertions was to treat the sores of the cancer on the cervix. This finding needs to be confirmed or otherwise in large patients with cervical cancer. In addition, and consistent with previous studies [30–32], the women stated that the cost of TM was inexpensive compared to conventional cancer treatments in the hospital.

Furthermore, even though all the women combined TM with conventional cancer treatment after their cervical cancer diagnosis in the hospital, four women out of 12 expressed the usefulness of TM and attributed their improved health condition to TM. The study recommends the development of a cervical cancer prevention education program that targets women’s reproductive and sexual health, spirituality, herbal intravaginal insertion, behavioral and lifestyle changes, as well as the provision of life empowerment opportunities to women. Such a program should include herbalists or TMPs, pastors, traditional birth attendants, and grandmothers as these individuals play crucial roles in the lives of women in Ghana.

### Study recommendations

The study found that TM is used for the treatment of cervical cancer. This finding has clinical implication for patient safety considering the mode and route of the treatment and location of the cancer. The study recommends clinicians assess patients diagnosed with cervical cancer for history of TM use, develop follow-up plans to monitor patients diagnosed with cervical cancer for TM use, and assess for possible effects of combined TM use and conventional cancer treatment and intervene to ensure patient safety.

The study identified several misconceptions (e.g., prayer heals cervical cancer, insertions of “balm” or ointment into the vagina heals cervical cancer). To dispel these misconceptions, the study recommends a cervical cancer prevention education program that targets young girls and women, herbalists or TMPs, pastors, traditional birth attendants, and grandmothers in Ghana.

The women were given TM for the treatment of cervical cancer without definitive diagnosis by the herbalist or TMPs. The study recommends that the Traditional Medical Practice Council in Ghana which has the mandate to license, regulate, and oversee the work of TM and CAM practitioners [33] should be proactive in the application of measures on how patients are diagnosed and managed. Such proactiveness will ensure that TMPs do not treat abnormal symptoms without a formal diagnosis in order to improve patient care. Research is also recommended to investigate the diagnostics practices of herbalists or TMPs for cervical cancer to inform appropriate and prescribe training for herbalists or TMPs in Ghana.

The study further recommends investigations on the identification of prescribed medicinal plants and analysis of their anti-cancer properties for the treatment of cervical cancer.

The Women inserted products into the vagina for the purpose of treatment of cervical cancer. To understand the scope of this practice and identify all the inserted products, research is recommended to identify all herbal intravaginal insertion products and confirm the practice of herbal intravaginal insertion for the treatment of cervical cancer in large patients with cervical cancer.

### Strengths and limitations of the study

This is the first qualitative study to describe the experiences of women with TM usage for cervical cancer treatment. The open-ended questions enabled the researchers to probe into the findings identified. The involvement of women in the acquisition and preparation of herbal products for cervical cancer treatment was described.

The limitations of the study include (1) Lack of data triangulation from herbalists or TMPs; (2) Small sample size appropriate for a qualitative inquiry but may not be generalizable to the entire Ghanaian cervical cancer patients; and (3) Study was limited to women in the Accra Metropolis and may not represent entire Ghanaian women. Despite these limitations, the study makes an important contribution to the literature on our understanding of how TM is used in the treatment of cervical cancer.

## Conclusions

The study identified that women consulted herbalists or TMPs first for the treatment of signs and symptoms of cervical cancer, the herbalists treated the women for cervical cancer without definitive diagnosis, and the main forms or types of TM the women used were HM in the form of sap (extracted from herbs, roots, leaves, and flowers) through the process of boiling and prayer as a treatment for cervical cancer. The routes through which HM was administered to effect healing of cervical cancer include oral (drinking), skin (bathing), and vagina orifice (douching).

## Supplementary Information

Supplementary Material 1

## Data Availability

The dataset supporting the conclusion of this article is included within the article and its additional files.

## Abbreviations

CAM: Complementary and Alternative Medicine
CTM: Chinese Traditional Medicine
GARH: Greater Accra Regional Hospital
GHS-ERC: Ghana Health Services Ethics Review Committee
HM: Herbal Medicine
NMIMR-IRB: Noguchi Memorial Institute for Medical Research Institutional Review Board
TM: Traditional Medicine
TMPs: Traditional Medicine Practitioners
USA: United States of America
