# Supplementary material for: Women’s experiences with traditional medicine for the treatment of cervical cancer: a qualitative study
Source: BMC Complement Med Ther. 2026 Mar 13;26:150. doi: 10.1186/s12906-026-05344-z (PMC13097700; doi:10.1186/s12906-026-05344-z)
Supplement: Supplementary file 1 — Supplementary Material 1 [file 12906_2026_5344_MOESM1_ESM.pdf]

Background Information and Interview Guide

Study Title: Women's experiences with traditional medicine for the treatment of cervical cancer in the Accra Metropolis

PI: Asakitogum Ayangba David

Address: School of Nursing and Midwifery, University of Ghana
adasakitogum@st.ug.edu.gh

Background Information

Participant Code Number: ……………………………………

1. Age:

2. Place of residence………………………………………………

3. Nationality ……………………………………………………..

4. Marital status: Married [ ]; Divorced [ ]; Never married [ ]; Separated [ ]

5. If married/divorced, is this the first or second marriage?

6. Age of first sexual encounter? …………………………….

7. Number of sexual partners you have had? …………………………….

8. Have you ever had a sexual partner who had multiple sexual partners?

9. Number of children………………………………………………….

10. Occupation ………………………………………………………

11. Level of education …………………………………………………

12. Languages spoken ………………………………………………….

13. Religion ………………………………………………………….

14. How long have you been diagnosed with cervical cancer? ....

15. Have you used traditional/herbal/indigenous medicine to treat your cervical cancer?

16. Are you still receiving treatment? Specify ……………………………………….

17. Do you smoke?...

18. Have you ever used a birth control method?

19. If yes to question 18 above, which type of birth control method did you use?

20. How long did you use this birth control method in question 19 above?

Interview Guide

The questions are open-ended questions developed according to the objectives of the study to guide the conduct of the interviews.

Women's Experiences with traditional interventions for cervical cancer

1. Please, share with me what informed your decision to use traditional/herbal/indigenous medicine to treat your cancer.

Probe: availability, affordability, efficacy

2. What did the traditional healer say was the cause of your disease?

Probe: poisoning, witches have to remove your womb, works of the enemies, spiritual, breaking taboo

3. Please, share with me the ways by which the traditional healer used to arrive at what was wrong with you.

Probe: Dancing and Chanting, Look into Mirror or Calabash, Tosh cowries or coin, Fasting and Prayers, Your feelings about it

4. Please, describe the kinds of treatment you received from the traditional healer to cure your cancer.

Probe: herbs, prayers, massage, witchcraft removal, experiences (pain, cost, work, support)

5. Share with me the results of using traditional medicine to treat your cancer.

Probe: worst, same status, psychological comfort, physical pain
